# Enhancing PM_2.5_ Prediction Using NARX-Based Combined CNN and LSTM Hybrid Model

**DOI:** 10.3390/s22124418

**Published:** 2022-06-11

**Authors:** Ahmed Samy AbdElAziz Moursi, Nawal El-Fishawy, Soufiene Djahel, Marwa A. Shouman

**Affiliations:** 1 Computer Science and Engineering Department, Faculty of Electronic Engineering, Menoufia University, Menouf 32952, Egypt; nawal.elfishawy@el-eng.menofia.edu.eg (N.E.-F.); marwa.shouman@el-eng.menofia.edu.eg (M.A.S.); 2 Department of Computing and Mathematics, Manchester Metropolitan University, Manchester M15 6BH, UK

**Keywords:** air quality prediction, PM_2.5_, NARX neural network, machine learning, CNN–LSTM

## Abstract

In a world where humanity’s interests come first, the environment is flooded with pollutants produced by humans’ urgent need for expansion. Air pollution and climate change are side effects of humans’ inconsiderate intervention. Particulate matter of 2.5 µm diameter (PM_2.5_) infiltrates lungs and hearts, causing many respiratory system diseases. Innovation in air pollution prediction is a must to protect the environment and its habitants, including those of humans. For that purpose, an enhanced method for PM_2.5_ prediction within the next hour is introduced in this research work using nonlinear autoregression with exogenous input (NARX) model hosting a convolutional neural network (CNN) followed by long short-term memory (LSTM) neural networks. The proposed enhancement was evaluated by several metrics such as index of agreement (IA) and normalized root mean square error (NRMSE). The results indicated that the CNN–LSTM/NARX hybrid model has the lowest NRMSE and the best IA, surpassing the state-of-the-art proposed hybrid deep-learning algorithms.

## 1. Introduction

During the last century, the human population on Earth has exploded [1]. Thus, for humanity’s survival and prosperity, rapid expansion in urbanisation, industry adoption, and transport systems development were inevitable. A direct consequence has been the unprecedented usage of natural resources such as fossil fuels and deforestation, resulting in the release of significant amounts of air pollutants into our host’s—the Earth’s—atmosphere. Air pollution is defined as the presence of pollutants in the atmosphere that damages humans’ health [2]. The damage inflicted by air pollution is not limited only to humans; it extends to all living creatures and the environment.

Moreover, recent research studies show that climate change is directly connected to air pollution [3]. The Earth’s atmosphere is afflicted by many contaminants produced by a plethora of anthropogenic sources, such as intense usage and dependence on transportation systems, house-heating systems, and energy generation via fossil fuel combustion, to name a few. This pollution negatively impacts human health, amplifies mortality rates in humans and other species living on Earth, and results in substantial global climate change [4].

The most significant six air pollutants (criteria air pollutants) as defined by the United States Environmental Protection Agency (US-EPA) are suspended particle matter (PM), nitrogen dioxide (NO_2_), ground-level ozone (O_3_), carbon monoxide (CO), sulphur dioxide (SO_2_), and lead (Pb) [5]. In this research, we focus on predicting suspended particulate matter, which are the fine particles found suspended in the atmosphere. The reported sources of PM are dust, forest fires, and man-made sources such as manufacturing processes and vehicle emissions, amongst others. There are two main types of PM, based on the approximate size of the particle. As defined by the World Health Organization (WHO) and US-EPA, PM_10_ particles have a diameter less than or equal to 10 μm (which includes PM_2.5_), whereas PM_2.5_ particles’ diameter is 2.5 μm or less [6,7]. Also, PM_1_, having a diameter ≤1 µm, is gaining more attention in recent research studies [8,9] although no limiting guidelines for PM_1_ have been published by WHO or US-EPA.

Of all the air pollutants mentioned above, fine particles (PM_2.5_ and PM_1_) are deemed the worst, affecting human lung function and worsening medical conditions such as asthma if exposure is longer than the standard period. This effect comes from the tiny PM_2.5_ that can invade the respiratory tract deeply, accumulating in and blocking fine blood vessels. All PM types are measured usually in µg/m^3^.

According to US-EPA, there are two types of standards: primary and secondary [10]. The primary standard is intended to protect the health of sensitive people such as children and the elderly, and people with respiratory health conditions. The secondary standard is aimed at protecting public welfare matters such as decreased visibility protection and buildings, animals, crops, and vegetation protection. PM_2.5_’s primary standard for one year, calculated as an annual mean over three years is 12 µg/m^3^. For a 24-h average calculated as the 98th percentile, averaged over three years, the primary and secondary standard is 35 µg/m^3^.

In a recent guideline published by the WHO in 2021 [11], there are two recommendations for PM_2.5_ air-quality guideline (AQG) levels: annual and short-term. Both recommendations use interim targets to introduce reductions of pollution levels in a gradual manner. The annual PM_2.5_ AQG targets 1 to 4 are 35, 25, 15, 10 µg/m^3^ respectively and the AQG recommended level is 5 µg/m^3^. The short-term (24-h) AQG level is defined as the 99th percentile (equal to 3–4 overexposure days per annum) of the annual distribution of 24-h average concentrations. The recommended short-term PM_2.5_ AQG targets 1 to 4 are 75, 50, 37.5, and 25 µg/m^3^ respectively, and the AQG specific level is 15 µg/m^3^.

Air pollution’s severe impact has driven the world to devise indices to assess air quality and determine the degree of safety for exposure amongst different groups of individuals [12]. Scientists have been developing methods to predict future air pollution levels, including chemical equations, physical simulations, and statistical models. Such models do not employ current advances in artificial intelligence practices and only apply physical, mathematical, and statistical methodologies. These models are limited when handling large datasets, leading scientists to use machine-learning methods to predict air quality [13,14,15]. Monitoring systems that used sensors to measure air pollutants concentration and stored the readings in large datasets enabled machine-learning scientists to exploit various algorithms for forecasting future air pollution levels [16]. Machine learning is utilised in various areas of modern society, and its first use in the environmental science domain dates to the 1990s. It has been applied in numerous environmental disciplines, including but not limited to ecological modelling, air pollution prediction, and weather forecasting [17]. Even with its broad application spectrum, the adoption of machine learning in environmental science has not prevailed as in other domains.

Nevertheless, as more data is being recorded about every aspect of the globe in our time, the attention on machine learning in the environmental field is increasing. Contrasted with classical statistical methods, machine learning gives better results because it has a better capacity to model complicated and nonlinear connections between data that exist in the natural world [18]. Due to the threats imposed by PM_2.5_, several attempts to predict its concentration in various regions using multiple methods have been conducted. This paper uses nonlinear autoregression with exogenous input (NARX) neural network with multiple configurations enhancing CNN–LSTM to predict PM_2.5_ concentration for the next hour with more accuracy. NARX was able to select the most effective subset of the features and pass them to CNN, which, along with dilation, was able to better map those features for LSTM timeseries prediction, to give better results than recent methods.

In particular, this paper’s key contributions are summarised as follows:

- Proposing an enhanced version of CNN–LSTM using NARX architecture.
- Evaluating multiple configurations of NARX using CNN–LSTM, LSTM, Extra Trees, and XGBRF.
- Comparing our work to both APNet [19] and NARX LSTM (d8, o1) [20] in terms of IA, it was found that the CNN–LSTM/NARX hybrid model produces better results than both.
- Executing our experiments on different cities in two separate locations located on distant continents (Beijing, China; Manchester, UK) and proving that our hybrid model can work well, regardless of the location.

The rest of this paper is constructed as follows. Section 2, “2. Related Work“, enlists the most recent and relevant published articles on the highlighted topic. Section 3, “3. Prediction Algorithms“, gives the essential knowledge regarding the algorithms used in our paper. Section 4, “4. Proposed Algorithm“, presents a detailed description of our proposal. Afterwards, in Section 5, “5. Performance Evaluation“, the evaluation metrics are introduced first, followed by a description of the used dataset and finally, the obtained results are presented and discussed in detail. Section 6, The “6. Conclusions” section summarises the outcomes and remarks from this research.

## 2. Related Work

As a result of the popularity and effectiveness of machine-learning and deep-learning methods, many studies use deep learning to predict PM_2.5_ or PM_10_. Here, the focus is to present recent studies that use CNN combined with LSTM to predict air pollutants, showing their advantages and their disadvantages. In addition, NARX’s recent studies are discussed to contrast their work to ours.

In 2018, Huang et al. [19] proposed APNet, a hybrid algorithm to predict PM_2.5_ by combining LSTM and CNN. They used 24 h of PM_2.5_, cumulated rain and wind speed to forecast PM_2.5_ for the next hour. They used the dataset in [21]. Their approach surpassed the exclusive use of CNN or LSTM and other baseline machine-learning algorithms. Various metrics were used for evaluation, including Pearson correlation coefficient, root mean square error (RMSE), mean absolute error (MAE), and index of agreement (IA). Although they proved the feasibility of their solution, the algorithm predictions did not follow the trend of PM_2.5_ pollution accurately. This inaccurate following is due mainly to the instability of PM_2.5_ pollution sources.

Qin et al. [22], in 2019, proposed a combined CNN–LSTM scheme to predict PM_2.5_ for the next 3 h using the past 24–72 h. They used CNN to feature data extraction spatially for multiple monitoring stations in one city (Shanghai). Then, the resultant feature map was fed to LSTM for timeseries prediction. Finally, an elastic net fine-tuned the results with the help of stochastic gradient descent to regularise constraints, fix network weights, and solve the over-fitting issue. RMSE and correlation coefficient evaluated their model. They used back propagation (BP), recurrent neural networks (RNN), CNN, and LSTM as a baseline for comparison. Their model can be used for processing input from many sites in a city. They have not verified their model to work in other cities.

Another study was carried out in 2020 to predict PM_10_ in various locations in Turkey [23]. Their data were collected in Istanbul between 2014 and 2018 to predict PM_10_ using 4-, 12-, and 24-h window sizes before the target hour. The study used many parameters to compare and optimise their work, including multiple window sizes and optimisers, and loss functions, and batch sizes. They used mean absolute error (MAE) and root mean square error (RMSE) to measure the performance. They combined data from meteorological and traffic sources and air pollution stations to compare the effectiveness of adding external sources for better air-quality prediction. Their proposal used a flexible dropout layer whose dropout rate depends on the window size. They used all the available data and features, which would incur a high computation cost and long execution time.

A multivariate CNN–LSTM model was introduced by [24] to forecast the next 24 h of PM_2.5_ concentration in Beijing, using data from the past week. CNN extracted air pollution features, whereas LSTM performed the timeseries forecast of the historical input. Univariant and multivariate editions of CNN–LSTM were examined vs. exclusive use of LSTM. RMSE and MAE were the evaluation metrics. Nevertheless, more metrics such as IA or R^2^ could have been used to confirm the correlation of the predicted values vs. actual values.

To select a subset of the history of pollutants and related atmospheric conditions, NARX was employed by [20]. They used a NARX neural network to apply LSTM and other algorithms for PM_2.5_ prediction in the next hour. They used multiple configurations and delays of the external inputs and 24 h of past PM_2.5_ to show the effect of using a subset of the data for prediction. The results show that using a subset gives better results and less training time. For evaluation, K-Fold was used by splitting the data into ten parts, then using one part as a test and the others as training data in a rotating style. This method is not optimal for timeseries problems as it includes training of the model with data that occurred after the test segment. This method could cause a data leak [25], as the model is trained with data from the future and then tested using past data.

This study focuses on leveraging the merits of CNN as a feature-mapping algorithm and the timeseries prediction capabilities of LSTM guided by the NARX neural network selection power to enhance the prediction accuracy of PM_2.5_. Our approach not only uses less training data by selecting certain past timesteps via NARX, but it also uncovers hidden patterns in data by using dilation in the CNN process. When compared to state-of-the-art methods that used the same dataset, and even another dataset of a city in a distant continent, our approach improved prediction results as measured by multiple metrics.

A summary of related work is presented in Table 1.

## 3. Prediction Algorithms

To show the effect of using CNN–LSTM with NARX, a brief introduction of each of the components used and the baseline models are presented.

### 3.1. Nonlinear Autoregression with Exogenous Input (NARX)

**Figure 1:**
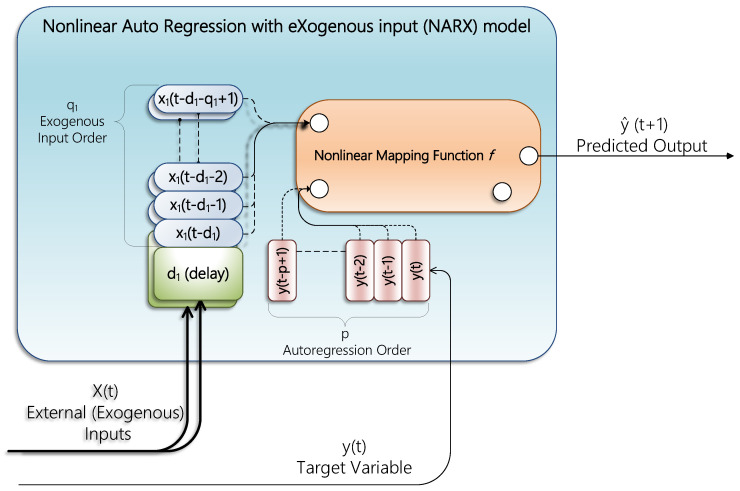
NARX model.

NARX is primarily utilised in timeseries analysis. It represents the nonlinear form of the autoregressive prediction model with external (exogenous) input. The autoregressive part of the model predicts output in a linear fashion based on earlier values. As a result, NARX connects the present value of a timeseries to preceding values of the series and the current and former values of the driving (external) series. Mapping input data to output can be done via a function. Frequently, that mapping is nonlinear, and any mapping function can be used, such as machine-learning techniques, Gaussian processes, neural networks, a mix of the preceding, or any other mapping function. NARX’s general concept is depicted in Figure 1 [26].

The model operates via selecting input features amongst consecutive timesteps t, and grouping former timesteps of external input order to be of length q each. Every input feature can be independently deferred by d timesteps. This model suggests choosing how many timesteps to include for each feature by order q and delaying them by d steps. Figure 1 illustrates that concept by incorporating one input feature $x_{1}$ using only $q_{1}$ timesteps (exogenous order) delayed by $d_{1}$. A shadow of another input in Figure 1 clarifies the idea of delay and order for multiple inputs. Likewise, the target data are stacked to represent autoregression of p timesteps (auto order). A Python library, fireTS, has been published [27] to enable using any scikit-learn [28] compatible regression to be the nonlinear mapping function for NARX. Generally, NARX is computed as in [29]:

(1) $$\hat{y}\left(t+1\right)=f\left(\begin{array}{c} y\left(t\right),\;y\left(t-1\right),\;y\left(t-2\right),\ldots ,y\left(t-p+1\right),\\ x_{1}\left(t-d_{1}\right),x_{1}\left(t-d_{1}-1\right),x_{1}\left(t-d_{1}-2\right),\ldots ,x_{1}\left(t-d_{1}-q_{1}+1\right),\\ \ldots ,\\ \;x_{m}\left(t-d_{m}\right),x_{m}\left(t-d_{m}-1\right),x_{m}\left(t-d_{m}-2\right),\ldots ,\;x_{m}\left(t-d_{m}-q_{m}+1\right) \end{array}\right)+e\left(t\right)$$

where $\hat{y}$ is the forecasted value; $f\left(.\right)$ represents any nonlinear mapping function; y is the target output at any timestep t; p is the length of target timesteps (autoregression order) specifying how many timesteps to use of the target for the prediction process; $x_{1},\ldots ,\;x_{m}$ are m external input features; $q_{1},\ldots ,\;q_{m}$ are the order associated with each of the exogenous inputs, controlling how many timesteps are captured for each input feature; $d_{1},\ldots ,\;d_{m}$ are the delays introduced to each m input feature; $e\left(t\right)$ is an error term, which is set to a random value, but, in our case, is set to zero.

It is also worth mentioning that NARX would prepare the input for the internal mapping function in a timeseries format. However, sometimes the internal function has some requirements to be met before processing the data. For example, LSTM would require input in 3-d format, and CNN would require data in a 4-d format.

NARX can also predict more steps in the future by using the predicted step and re-inserting it into the mapping function to get the next predicted step. NARX has been used by researchers in air-quality prediction [20], evaluating visibility range on air pollution [30], glucose level prediction [27], and data calibration [31]. The main advantage of NARX is that any nonlinear regression function can be used to perform regression on timeseries problems and that there is flexibility in choosing how much history to use. Also, compared to other recurrent networks, NARX converges quicker and takes fewer training cycles [32].

### 3.2. 1-D Convolution Neural Network (1-D CNN)

A convolution neural network uses a convolution operation through a filter to extract patterns or features from input data. CNN is well-known in the image analysis domain. Nevertheless, CNN has multiple network structures, including 1D CNN, 2D CNN, and 3D CNN [33]. 1D CNN can be efficiently used in timeseries analysis [34], 2D CNN is frequently applied in text and image recognition [35], and 3D CNN is employed in video data recognition and medical images analysis [36]. Therefore, 1D CNN is implemented to enhance this research’s results further. A simplified view of how 1D CNN works follows.

**Figure 2:**
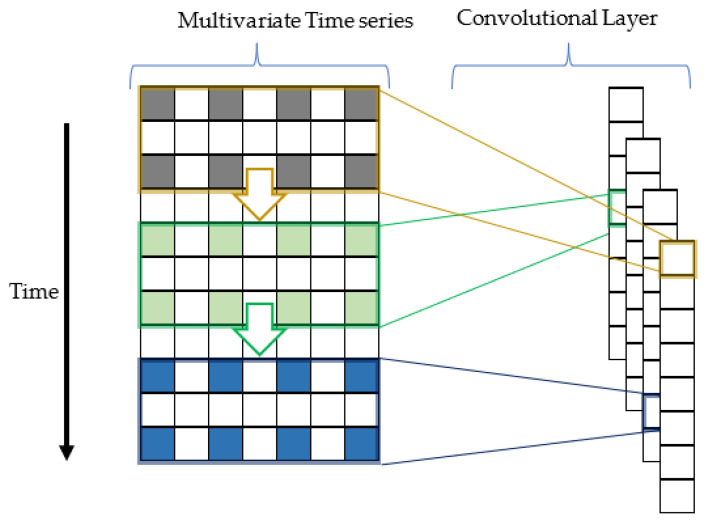
1D CNN process.

The left of Figure 2 represents the multidimensional input timeseries data (features + target), which is convoluted from top to bottom, as shown by the coloured arrows in Figure 2, and the coloured rectangles represent multiple filters. Each filter applies convolution that reduces dimensionality from the input to the convolutional layer. The filter uses dilation to select only coloured cells within each filter instead of all cells. This dilation effectively expands the filter size by inserting holes between adjacent elements. This way, a wider field of view is obtained at the same computational cost. CNN can be combined with LSTM [19,23,24,37,38] or support vector machine (SVM) [39]. CNN acts as a feature mapper to detect patterns inside the data.

### 3.3. Long Short-Term Memory (LSTM)

Timeseries studies are, in many cases, done best by the LSTM algorithm. It accepts not only the current input but also preceding outcomes. LSTM works by utilising the outcome at time $\left(t-1\right)$ as the input at time (t), in conjunction with the new input at time (t) [40]. Therefore, contrary to the “feedforward networks”, ‘memory’ is accumulated inside the network. This feature is crucial to LSTM as constant information exists about the past sequence itself, not only the outputs [41]. Air contaminants fluctuate over time, and long-term exposures to PM_2.5_ are associated with health risks. Throughout lengthy periods, it is evident that the most accurate upcoming air pollution predictor is the earlier air pollution [42].

**Figure 3:**
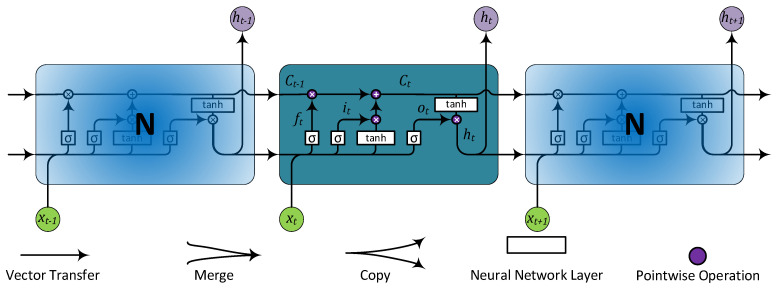
LSTM RNN elemental network structure.

LSTM is a good model for timeseries prediction because it sustains errors in a gated cell. LSTM is illustrated in Figure 3.

The following equations describe the LSTM forward training process [43]:

(2) $$f_{t}=\sigma \left(W_{f}\cdot \left[h_{t-1},x_{t}\right]+b_{f}\right)$$

(3) $$i_{t}=\sigma \left(W_{i}\cdot \left[h_{t-1},x_{t}\right]+b_{i}\right)$$

(4) $$o_{t}=\sigma \left(W_{o}\cdot \left[h_{t-1},x_{t}\right]+b_{o}\right)$$

(5) $$C_{t}=f_{t}*C_{t-1}+i_{t}*tanh\left(W_{C}\cdot \left[h_{t-1},x_{t}\right]+b_{c}\right)$$

(6) $$h_{t}=o_{t}*tanh\left(C_{t}\right)$$

where $i_{t}$, $f_{t},$ and $o_{t}$ are activation functions of the input gate, forget gate, and output gate, respectively; $h_{t}$ and $C_{t}$ are the activation vectors for each memory block and cell, respectively; and b and W are the bias vector and weight matrix, respectively. Also $tanh\left(\cdot \right)$ represents the tanh function defined in Equation (7), and $\sigma \left(\cdot \right)$ is the sigmoid function, specified in Equation (8).

(7) $$tanh\left(x\right)=\frac{e^{x}-e^{-x}}{e^{x}+e^{-x}}$$

(8) $$\sigma \left(x\right)=\frac{1}{1+e^{-x}}$$

Since LSTM uses sigmoid and tanh functions, they usually require input data to be normalised from 0 to 1 to get accurate results.

### 3.4. Extra Trees (ET)

Extra Trees is a machine-learning methodology that solves classification and supervised regression problems via a tree-based ensemble method. Its core idea is to build ensembles of unpruned decision trees using the top-down technique. It constructs completely randomised trees with constructions distinct from the learning sample. The Extra Trees algorithm has been developed to compensate for the high variance errors resulting from using a single decision tree.

All decision-tree-based methods, including boosted versions, cannot predict values outside the training data range [44], so they cannot extrapolate.

### 3.5. Random Forests in XGBoost (XGBRF)

Both XGBoost and Random Forest are well-known decision tree-based algorithms. XGBoost is a boosting algorithm, while Random Forest is a bagging algorithm. As a result, their combination is known as a hybrid ensemble learning model. Random Forest replaces the decision tree as the basic estimator in the GBRF model [45]. The XGBRF regressor is an improved version of the XGBoost regressor.

The XGBRF trains Random Forest decision trees instead of the gradient-boosting decision trees employed directly by the XGBoost regressor and achieves good accuracy on various datasets. The XGBRF takes advantage of both the XGBoost and the Random Forest models to provide high stability and accuracy and avoid the overfitting problem.

Gradient-boosted models, including gradient-boosted decision trees, are trainable with XGBoost or Random Forests. This training process is feasible since they share the same model inference and representation techniques; however, their training procedures are distinct. XGBoost can use Random Forests either as a basic model for gradient boosting or as a training target. The focus of XGBRF training is on isolated random forests. This technique is a scikit-learn [28] wrapper introduced in the open-source, and still experimental, XGBoost package [46], which implies that the interface can be altered. XGBRF has been used by many studies such as [20,47].

## 4. Proposed Algorithm

**Figure 4:**
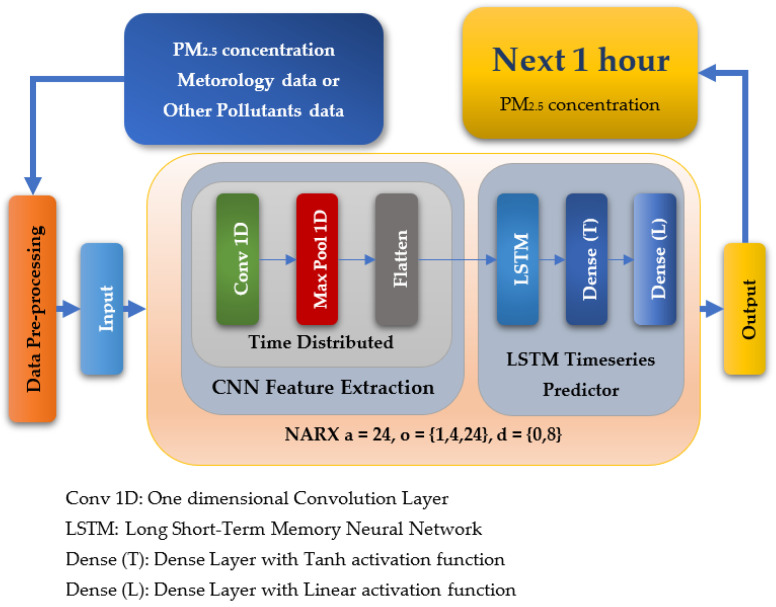
An overview of CNN–LSTM NARX proposed layers.

Our proposal wraps CNN–LSTM with NARX architecture. As shown in Figure 4, selected input features are pre-processed, removing rows containing invalid data and normalising them as required by the guest nonlinear function CNN–LSTM. Data is split gradually to feed the algorithm by a growing amount of training data in each iteration. It preserves the timeseries relationship by testing only future data using the old training history described in Section 5.2.3, “5.2.3. Data Preprocessing before Feeding to ML Algorithms”. NARX then refines input to CNN–LSTM using auto-order, exogenous order, and delay. CNN remaps features using convolution and dilation and feeds them to an LSTM neural network, which builds a timeseries predictor that learns the temporal relation between features and target.

Our proposed CNN–LSTM NARX architecture can be illustrated in Algorithm 1:
**Algorithm 1:** CNN–LSTM NARX Architecture Steps
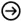
Input:Exogenous input features (meteorological data or other air pollutants) and one auto-input feature PM_2.5_.
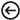
Output:PM_2.5_ for the next hour
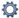
Processing:
First, preprocessing is done where data are normalised, and invalid data are removed.Data are divided into two sets (training/testing), where training sets always occur before testing sets.For training and testing, NARX selects a specified number of (PM_2.5_) history hours as defined by the autoregression parameter for CNN–LSTM and takes the definite timesteps for the exogenous input features as demanded by the exogenous order parameter, presenting the specific delay determined by the exogenous delay parameter of NARX.As CNN only accepts data in 4-D, reshaping is done before applying convolution with dilation.Each layer in CNN (Conv 1D, Max Pool 1D, Flatten) is wrapped in a time-distributed layer applying convolution for each timestep in the data.LSTM takes input from a flattened layer to perform learning; then, a tanh dense layer reduces output, which is further reduced by a linear dense layer to PM_2.5_ output.After training is done, testing data is applied to produce predetermined predictions.The overall system is evaluated using various metrics.

## 5. Performance Evaluation

### 5.1. Validation Metrics

To evaluate the prediction model’s performance and uncover any possible association between the forecast and actual values, the following metrics are calculated for our experimentations.

#### 5.1.1. Coefficient of Determination R^2^

This metric estimates the correlation between actual and projected values. It is determined as in [48]:

(9) $$R^{2}={\left(\frac{\sum_{i=1}^{n}\left(P_{i}-\bar{P}\right)\left(A_{i}-\bar{A}\right)}{\sqrt{\sum_{i=1}^{n}{\left(P_{i}-\bar{P}\right)}^{2}\sum_{i=1}^{n}{\left(A_{i}-\bar{A}\right)}^{2}}}\right)}^{2}$$

where *n* is the number of data items; $P_{i}$ and $A_{i}$ are the projected and actual values, in that order; $\bar{P}$ and $\bar{A}$ denote the mean of the projected and actual value of the pollutant, respectively.

$R^{2}$ is a descriptive statistical index. Hence, it has no unit of measurement or dimensions, and it ranges from 0 (no correlation) to 1 (complete correlation).

#### 5.1.2. Index of Agreement (IA)

A standardised measure model forecasting error with values between 0 and 1; *IA* was proposed in [49]. This metric is termed by:

(10) $$IA=1-\frac{\sum_{i=1}^{n}{\left(\left|P_{i}-A_{i}\right|\right)}^{2}}{\sum_{i=1}^{n}{\left(\left|P_{i}-\bar{A}\right|+\left|A_{i}-\bar{A}\right|\right)}^{2}}$$

where *n* is the records count; $P_{i}$ and $A_{i}$ are the projected and actual measurements, respectively; $\bar{P}$ and $\bar{A}$ symbolise the projected mean and actual mean value of the target, in turn.

In this dimensionless metric, 1 represents a total agreement, and 0 represents no agreement. It can identify additive and proportional differences in actual and projected means and variances, but it is too sensitive to extreme values owing to squared differences.

#### 5.1.3. Root Mean Square Error (RMSE)

*RMSE* computes the mean for the squared differences between predicted and actual values and then takes the square root of the result. It is calculated as in [48]:

(11) $$RMSE=\sqrt{\frac{\sum_{i=1}^{n}{\left(P_{i}-A_{i}\right)}^{2}}{n}}$$

where *n* is the samples count; $A_{i}$ and $P_{i}$ are the actual and predicted data, in that order.

*RMSE* has the identical measurement unit of the forecasted or real values, namely μgm3. The less *RMSE* value, the better the performance of the prediction model.

#### 5.1.4. Normalised Root Mean Square Error (NRMSE)

Normalising *RMSE* has many ways. One way divides *RMSE* by the difference between the maximum and the minimum of the actual value. Comparison of models or datasets with distinct scales is better achieved through *NRMSE*. Its computation is done via [50]:

(12) $$NRMSE=\frac{RMSE}{Max\left(A_{i}\right)-Min\left(A_{i}\right)}$$

### 5.2. Data Description and Preprocessing

#### 5.2.1. Beijing, China Dataset

The dataset utilised was obtained from air pollution, and meteorological data for Beijing, China, between 2010 and 2014 [21] and was published in the machine-learning repository of the University of California, Irvine (UCI). The dataset includes hourly data of a variety of meteorological conditions, including (pressure) hPa, (temperature, dew point) °C, (cumulated wind speed) m/s, combined wind direction, (cumulated snow) hours, and (cumulated rain) hours. It also contains the PM_2.5_ concentration in micrograms per cubic metre μgm3. All rows with missing values in the PM_2.5_ column were removed, and columns specifying the record time were eliminated. The dataset statistics before preprocessing are presented in Table 2.

#### 5.2.2. Manchester, UK Dataset

This dataset was compiled from the official website of the Department for Environment Food & Rural Affairs (DEFRA) [51] in the UK. It represents meteorological and air pollutants concentrations for Piccadilly station, Manchester, UK, from 2015 to 2019. It comprises the average hourly data of a variety of meteorological conditions, including (modelled wind direction—M_DIR) in ° degrees, (modelled temperature—M_T) °C, (modelled wind speed—M_SPED) m/s. It also covers hourly average concentrations of some air pollutants, including (PM_2.5_, NO, NO_2_, and O_3_) in μgm3.

Table 3 shows the statistics of the dataset before any processing. Because PM_2.5_ is measured as weight in a unit of volume, it is always a positive value or zero; hence clearing negative values is necessary. Also, there is some missing data in PM_2.5_ and other features, which implies the need for imputation to run machine-learning algorithms.

**Figure 5:**
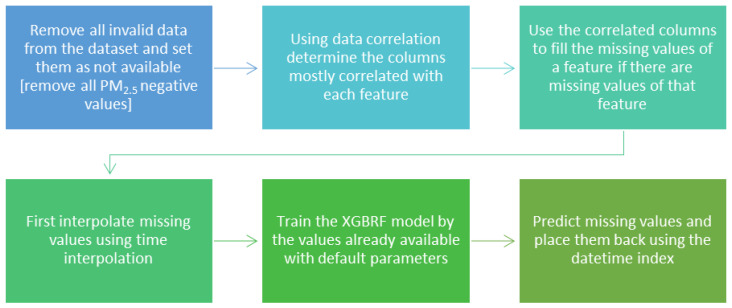
Imputation flowchart for every feature in Piccadilly station, Manchester, UK.

Figure 5 illustrates a flowchart of the imputation process applied to impute every feature in the dataset.

**Figure 6:**
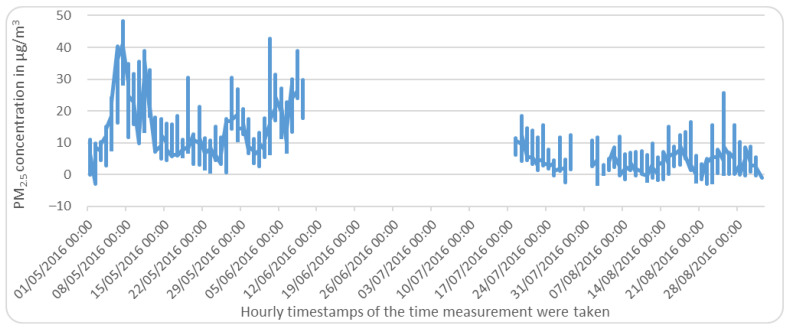
Sample of data from 1 May 2016 to 1 September 2016 data of Piccadilly station, Manchester, UK, before imputation.

**Figure 7:**
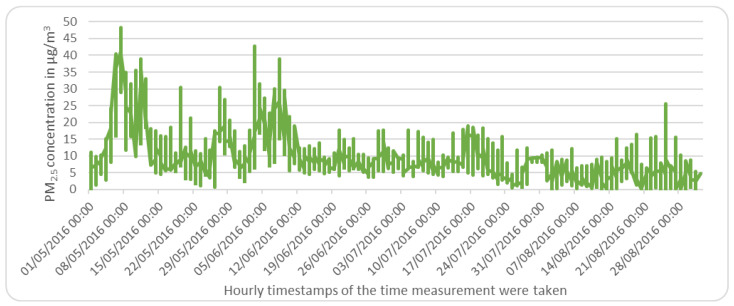
Sample of data from 1 May 2016 to 1 September 2016 data of Piccadilly station, Manchester, the UK, after imputation.

A comparison of a sample of the data before and after imputation is illustrated in Figure 6 and Figure 7, respectively.

Table 4 shows the statistics of the dataset after the imputation process. The minimum and maximum values are the same as the original dataset, and most other statistics are very close to the original dataset values.

#### 5.2.3. Data Preprocessing before Feeding to ML Algorithms

**Figure 8:**
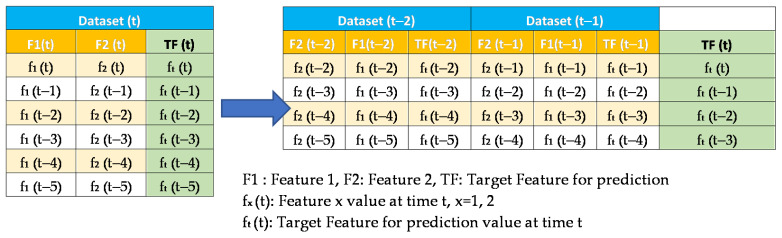
A sample dataset showing how data shifting is done for two look-back hours.

**Figure 9:**
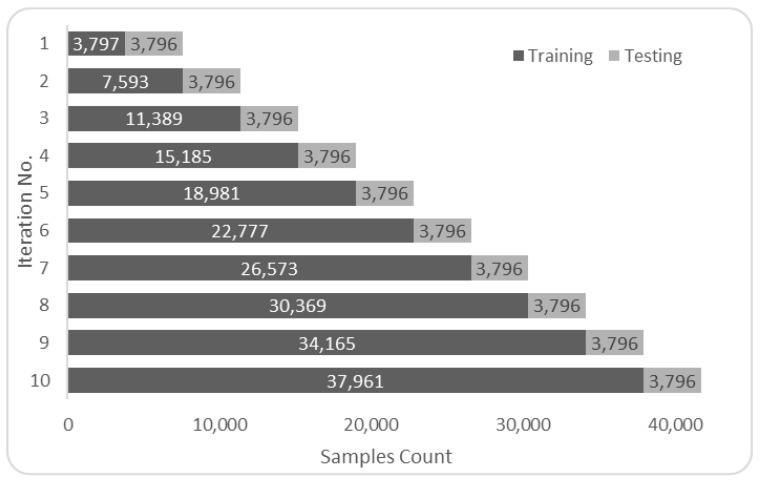
Training vs. testing in timeseries split cross-validation *n*=10 for the Beijing dataset.

**Figure 10:**
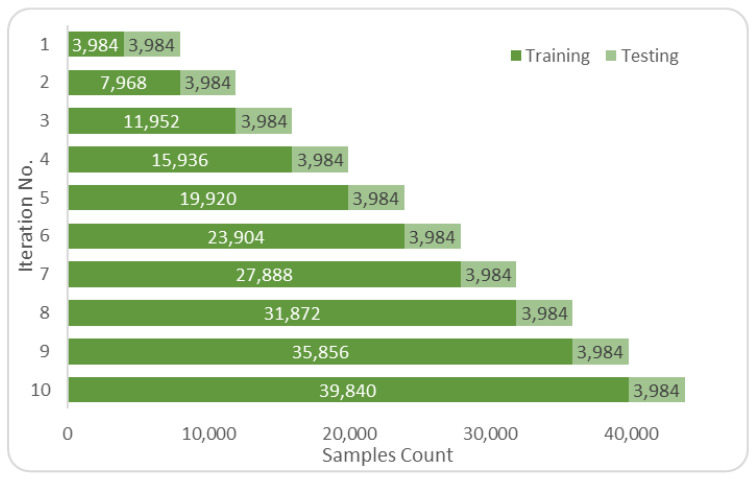
Training vs. testing in timeseries split cross-validation *n*=10 for the Manchester dataset.

The datasets were turned into a timeseries suitable form before being employed in any selected prediction algorithms [52]. Data from the previous 24 h were used to forecast PM_2.5_ for the upcoming hour. This choice has been made to be able to compare our work to others who used the same amount of look-back hours with the same dataset [19,20]. The transition was accomplished by moving recordings up by 24 places. These data were then inserted as columns after the current dataset, and the procedure was iterated recursively to produce the following structure: dataset (t−*n*), dataset (t−*n*−1), …, dataset (t−1), target feature (t) as the sample shown in Figure 8. The target feature shown in Figure 8 in the rightmost column of the right table uses past values of itself and other features of the past. It can be noted that the shift operation reduces the number of records by the number of past values or look back value used, 2 in this case. This form was employed in algorithms not using NARX. The training and testing samples were split using K-Fold adapted to handle timeseries situations and avoid data leaks [25]. The sampling was done as shown in Figure 9 and Figure 10.

### 5.3. Results Analysis and Discussion

The proposal was executed on a laptop equipped with an eight-core Intel processor core i9-9980HK CPU @ 2.40GHz- hyperthreading enabled-aided by 32 GB of DDR4 RAM and GeForce RTX 2060. The laptop was not entirely dedicated to the experiments, yet light background work was carried out mostly to ensure training time measured in Table 5 and Table 6 was not affected drastically by those tasks. Python 3.8 was used in all our experiments.

**Figure 11:**
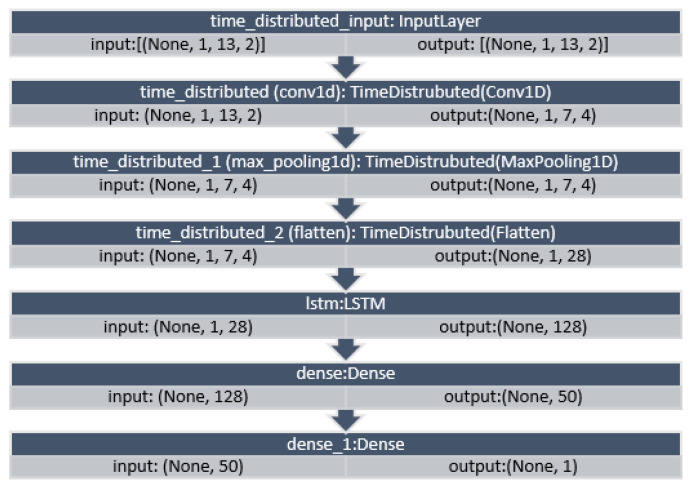
CNN–LSTM layers for the seventh iteration with (d0, o1) for the Beijing dataset.

**Figure 12:**
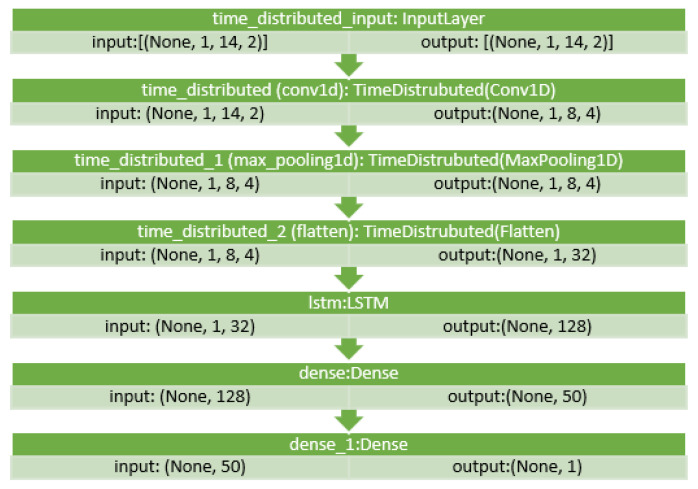
CNN–LSTM layers for the seventh iteration with (d0, o1) for the Manchester dataset.

Figure 11 and Figure 12 show the layers configuration of CNN–LSTM with NARX (d0, o1) on the seventh iteration as produced by Python for Beijing and Manchester, respectively. The seventh iteration was chosen to represent the average case as it has enough training data but not the whole training data as the last iteration.

As for parameters, CNN had three layers: 1.—Conv1D with a dilation rate of 6 and group 2 with 4 filters and a kernel size of 2; 2.—the maximum pooling layer had a size of 1; 3.—the flatten layer. LSTM was composed of three consecutive layers: 1.—an entry layer (128 nodes); 2.—a hidden intermediate layer (50 nodes); and 3.—a final layer (one node). LSTM used tanh as an activation function and utilised the adaptive moment estimation (Adam) optimiser to minimise the loss function (MAE) and a batch size of (72) with (25) epochs. The LSTM arrangement was used in [20,53]. Each other parameter in each algorithm used was the default as specified by the API of scikit-learn [54]. NARX parameters were 24 for PM_2.5_ auto-order and four permutations of exogenous delay (ed) and exogenous order (eo) for all features on all algorithms, specifically (0,1), (0,4), (0,24), and (8,1). To shorten the names and because the same delay and order are applied for all exogenous inputs, the following figures use the NARX version with the name (d*x*, o*y*), where *x* is the exogenous delay and *y* is the exogenous order.

All tests were executed in parallel on all central processing unit (CPU) cores to improve speed. LSTM and CNN–LSTM were run using GPU to speed up the training process. The subsequent figures show 72-h-sample timesteps forecast via our tests versus real values in the seventh iteration for each dataset.

**Figure 13:**
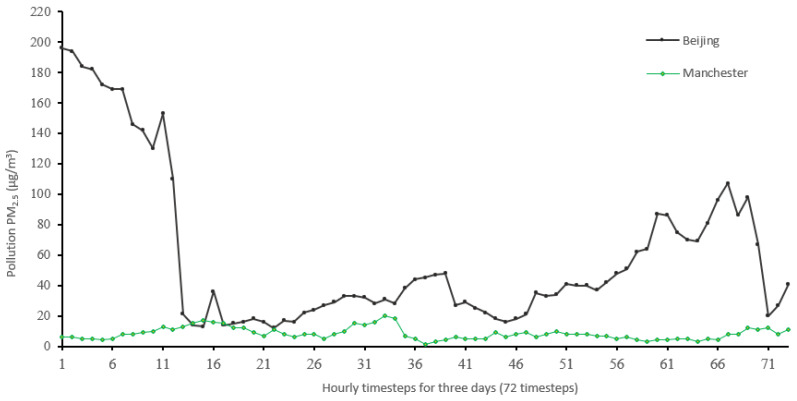
Real PM_2.5_ data of part of the seventh iteration results comparing the Beijing vs. Manchester datasets ranges.

To compare the ranges of PM_2.5_ in Beijing and Manchester, Figure 13 plots the real values used in the comparison shown in following figures.

**Figure 14:**
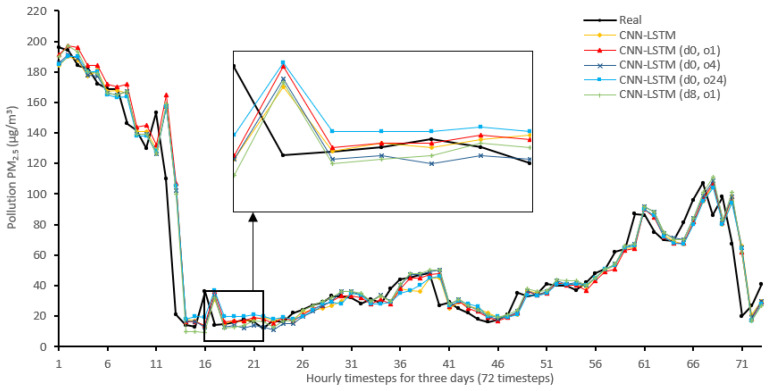
Real vs. CNN–LSTM and its NARX variants in part of the seventh iteration results for the Beijing dataset.

**Figure 15:**
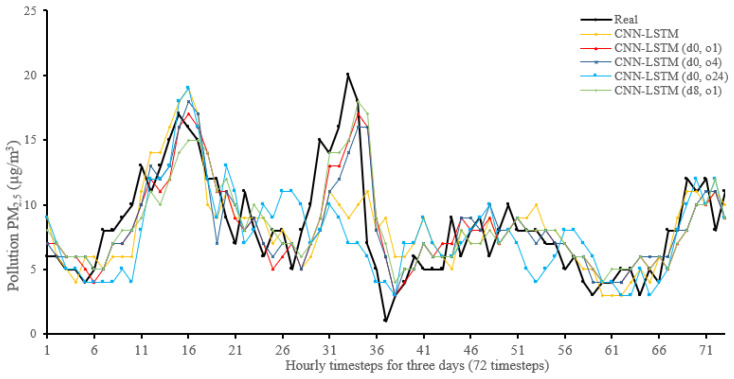
Real vs. CNN–LSTM and its NARX variants in part of the seventh iteration results for the Manchester dataset.

**Figure 16:**
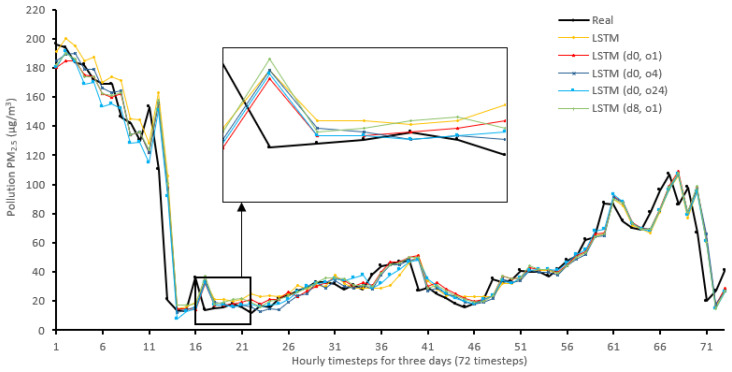
Real vs. LSTM and its NARX variants in part of the seventh iteration results for the Beijing dataset.

**Figure 17:**
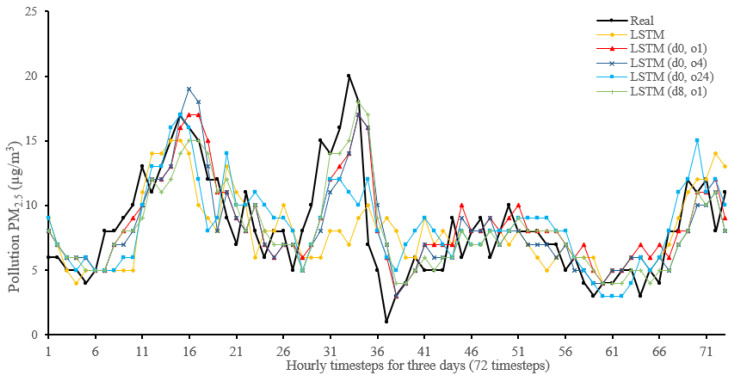
Real vs. LSTM and its NARX variants in part of the seventh iteration results for the Manchester dataset.

**Figure 18:**
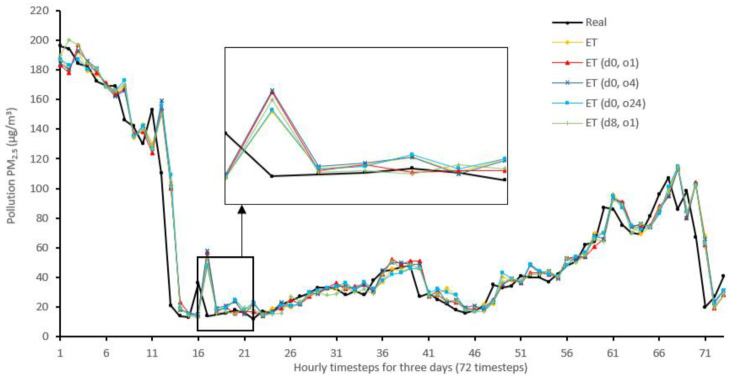
Real vs. Extra Trees and its NARX variants in part of the seventh iteration results for the Beijing dataset.

**Figure 19:**
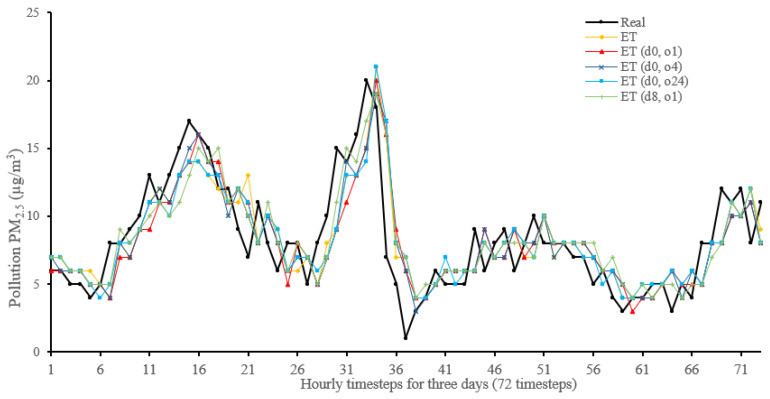
Real vs. Extra Trees and its NARX variants in part of the seventh iteration results for the Manchester dataset.

**Figure 20:**
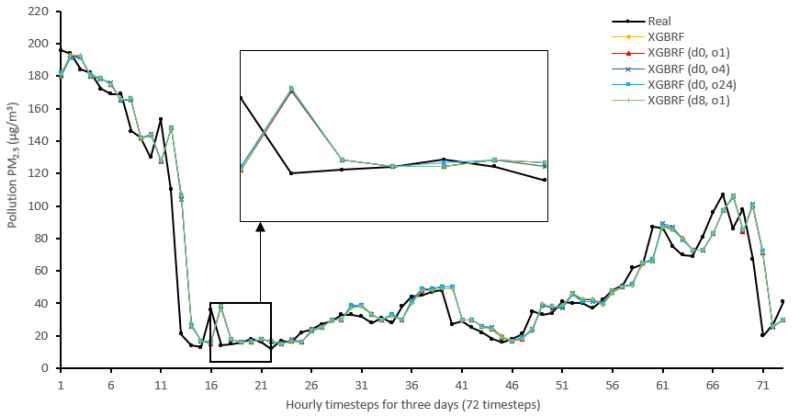
Real vs. XGBRF and its NARX variants in part of the seventh iteration results for the Beijing dataset.

**Figure 21:**
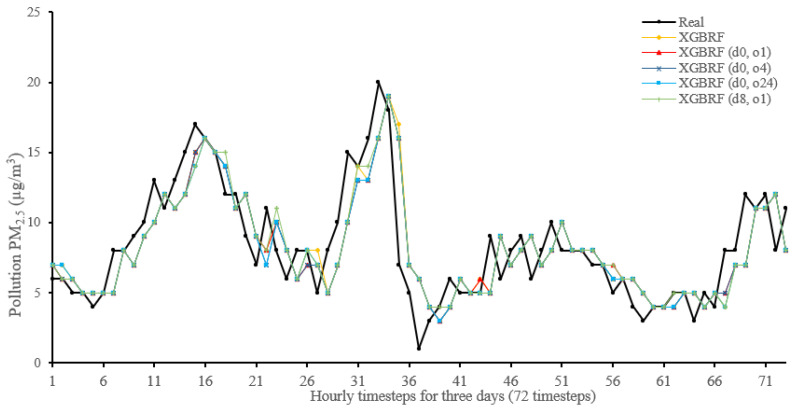
Real vs. XGBRF and its NARX variants in part of the seventh iteration results for the Manchester dataset.

Figure 14, Figure 15, Figure 16, Figure 17, Figure 18, Figure 19, Figure 20 and Figure 21 compare real PM_2.5_ values and their predicted counterpart using NARX and non-NARX algorithms during three days of the seventh iteration of the Timeseries Split K-Fold for each dataset. The data points show a slight time-shift between prediction and actual data. This shift is usually caused by most algorithms being greatly affected by the last value of the target more than other inputs in the training process.

Table 5 and Table 6 illustrate metrics results averaged over ten timeseries k-fold iterations for each dataset. The arrow next to each metric shows which direction gives the better result. The upward direction means the higher, the better; and the downward direction means the lower, the better. Numbers backgrounds have been done as a heat map where greener is better and redder is worse. Best values are bold and underlined with a single line. The worst values are double-underlined and italic. Evaluation metrics employed were R^2^, IA, RMSE, and NRMSE. Offline training duration (T_tr_) was calculated as the difference between two timestamps before and after training by Python. To further shorten the NARX variation name, after each non-NARX algorithm, the NARX version is denoted by (d*x*, o*y*), where *x* is the delay applied to all exogenous inputs and *y* is the order applied to all exogenous inputs.

Average results illustrated in Table 5 and Table 6 are depicted visually using Figure 22, Figure 23, Figure 24, Figure 25, Figure 26, Figure 27, Figure 28, Figure 29, Figure 30 and Figure 31, respectively, to ease comparison. The worst-value bar is coloured light red, whereas the best is coloured in light green. The figure has been sectioned to group each algorithm with its NARX variants.

Generally, all methods give good results in R^2^ and IA above 0.92 and 0.97 for the Beijing dataset, in turn, and 0.70 and 0.89 for the Manchester dataset, in that order. As NARX allows for selecting how much exogenous input and delay is to be used in the training and prediction process, the results differ according to these settings. Using NARX with CNN–LSTM gives the best results almost in all variations and across all metrics, especially with low external order (o1, o4), as the overview and close view in Figure 14 and Figure 15 illustrate. Also, in LSTM, as the Figure 16 general and zoomed window and Figure 17 show, better results are obtained with lower external orders (o1, o4). This effect is probably due to the memory element used in LSTM, which gets misled if fed an extended amount of data from external inputs, which it has already learnt about from previous training. In addition, in the case of CNN, the dilation used along with convolution did capture a hidden relationship between input elements leading to an even better prediction than the mere usage of LSTM at the cost of more processing time. Using NARX usually introduces less processing time in lower external orders, as in the case of ET and XGBRF. In the case of CNN–LSTM and LSTM, there is not much speed gain because of the low dimensionality of the data [55]. GPU usage is a must because convolution in CNN–LSTM with grouping is only supported by GPU implementation in TensorFlow [56].

For the Manchester dataset, RMSE values are generally low because of the low mean (10.42) and standard deviation (9.73) of PM_2.5_ in the dataset. In addition, the fact that there are few sharp transitions from low values to high values or vice versa, as shown in Figure 15 (change is from 20 to 1), as well as the shift that exists at much of the results, would cause that difference to be low, mostly. On the other hand, in the Beijing dataset, transitions are much sharper, as in Figure 14, from (153 to 21) along with the shift would cause higher error rates. Table 7 shows an excerpt from the Beijing dataset when the sharp transition happened, conforming to an increase in cumulated wind speed, especially in the northern direction. As described in [21], northerly wind decreases PM_2.5_ substantially in all seasons.

In contrast with Extra Trees and XGBRF, the more external input order, the better prediction results it gets, see Figure 16, Figure 17, Figure 18 and Figure 19. This result is primarily because of how these systems work as they build decision trees of the input currently present, and no memory element exists. Additionally, it can be noted from Figure 18 that there is little influence of using NARX on XGBRF. A possible reason is the low randomness of XGBRF and its low response to external variables.

Table 8 and Table 9 represent output statistics of the seventh iteration of prediction, including statistics about training and testing sets. Due to the shifts introduced by NARX and to align all predictions with real data, testing was cut from 3796 to 3722 rows (Beijing) and from 3984 to 3960 (Manchester).

As the previous tables indicate, the best algorithm in green CNN–LSTM NARX (d0, o1) for the Beijing dataset and CNN–LSTM NARX (d0, o4) for Manchester gives the closest output statistics to the statistics of the testing set except for the data towards the maximum. It can also be noted that the red numbers have gone below the limit of PM_2.5_, which is 0. This result indicates the ability of CNN–LSTM and LSTM to extrapolate or go beyond the limits of the training and testing data. The delay in the Beijing dataset’s CNN–LSTM (d8, o1) resulted in extremities in minimum and maximum (−15 less than 0 and 403 more than all other CNN–LSTM or LSTM variants but still less than the testing maximum). On the other hand, Extra Trees and XGBRF tend to interpolate and not go beyond the training limits. In Table 10 and Table 11, most maximum values in bold purple were close to the testing maximum except for the underlined cases, which are more than the testing maximum but still less than the training maximum. The minimum (marked by bold blue) tends to produce larger values than the testing minimum but never less.

Using CNN–LSTM with NARX and setting exogenous order to 1 or 4 with no delay gives better results than other methods. Moreover, in terms of RMSE our results for the Beijing dataset are better than APNet [19] (22.56670 vs. 24.22874, 7.36% error reduction) and NARX LSTM (d8, o1) [20] (22.56670 vs. 23.64560, 4.78% error reduction). In addition, for the Beijing dataset, CNN–LSTM with NARX reduced RMSE by 12.23% from the XGBRF baseline (22.56670 vs. 25.32772). Moreover, for the Manchester dataset, CNN–LSTM with NARX reduced RMSE by 5.41% from the XGBRF baseline (4.40502 vs. 4.64339). Although these improvements are not of great magnitude, they would make an increasing difference as more steps in the future depend on the next prediction step when using the recursive method. This improvement is important because the system will probably use the predicted next hour to build on and get the second hour in the future, the third, and other future steps.

## 6. Conclusions

In this work, an enhancement of PM_2.5_ prediction accuracy was proposed and evaluated using a combination of CNN and LSTM based on NARX. The experiments involved using a 24-h period of PM_2.5_ concentration in conjunction with meteorological features for Beijing and Manchester to predict the next hour’s concentration of PM_2.5_. Using CNN–LSTM and dilation and grouping data introduced better results than all tested methods, especially with low exogenous order and no delay. Our proposed enhancement produced better results than two state-of-the art methods on the same dataset. These methods are APNet (7.36% error reduction) and LSTM_NARX (d8, o1) (4.78% error reduction) for the Beijing dataset. An examination of predictions output statistics proved our enhancement to be the closest to the testing statistics and showed CNN–LSTM extrapolation capabilities.

## 7. Future Work

This research can be further expanded to include other data related to air pollution such as traffic data, which probably contributes even more to prediction than meteorological factors. In addition, more timesteps could be predicted in the future (24, 72 h for example) while studying the effects of using NARX in various future prediction methods, including direct and recursive methods. Combining ML methods with a physical model is another way to improve prediction performance in the future. As noted, there are many parameters used in this hybrid, and various methods are potential candidates to explore that domain and optimise those parameters, including but not limited to genetic algorithms and swarm optimisation methods, amongst others.

## Figures and Tables

**Figure 22:**
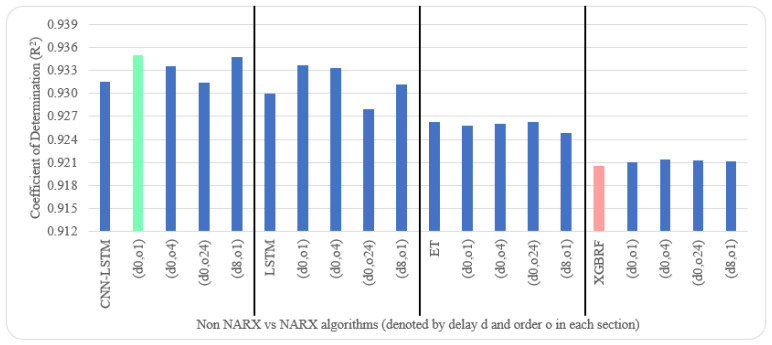
Evaluation results of non-NARX and NARX in terms of coefficient of determination for the Beijing dataset.

**Figure 23:**
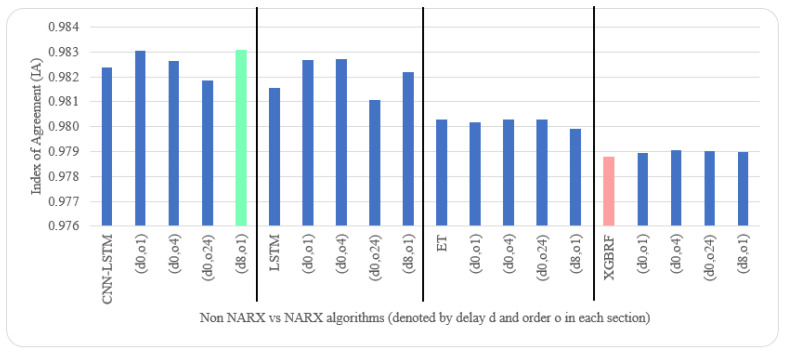
Evaluation results of non-NARX and NARX in terms of index of agreement for the Beijing dataset.

**Figure 24:**
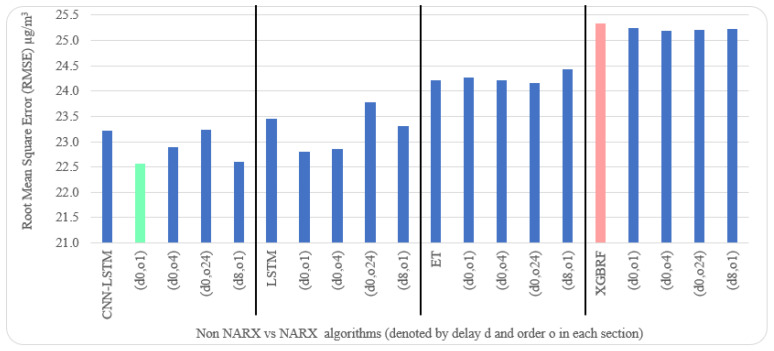
Evaluation results of non-NARX and NARX in terms of root mean square error for the Beijing dataset.

**Figure 25:**
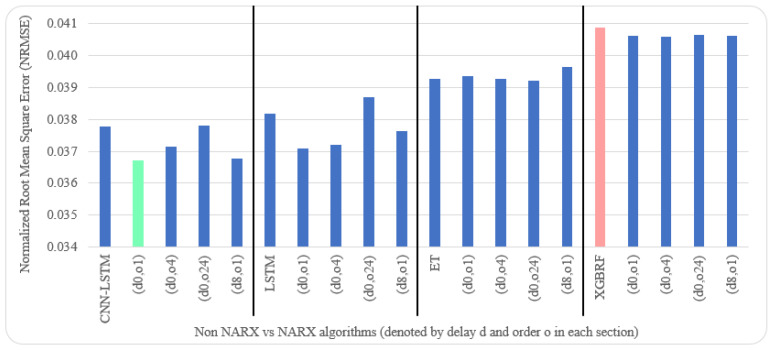
Evaluation results of non-NARX and NARX in terms of normalised root mean square error for the Beijing dataset.

**Figure 26:**
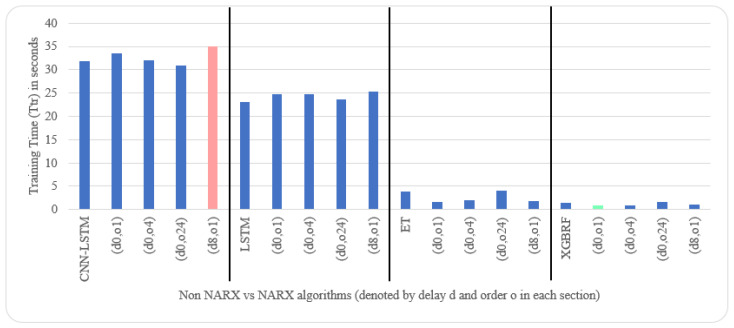
Evaluation results of non-NARX and NARX in terms of offline training time for the Beijing dataset.

**Figure 27:**
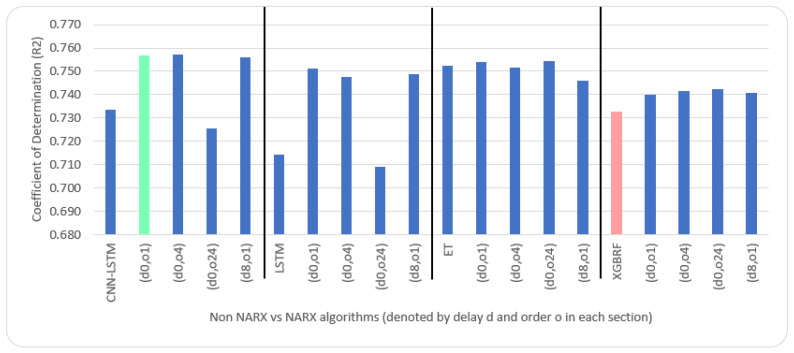
Evaluation results of non-NARX and NARX in terms of coefficient of determination for the Manchester dataset.

**Figure 28:**
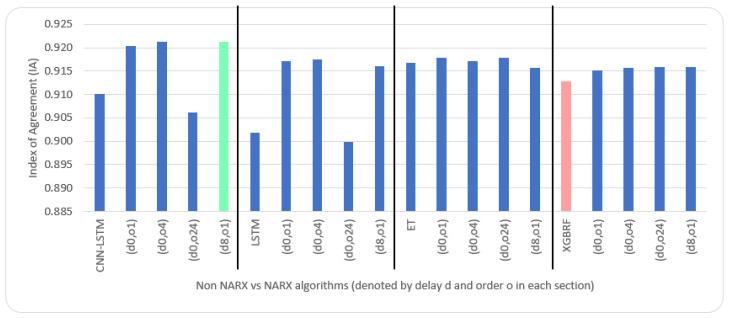
Evaluation results of non-NARX and NARX in terms of index of agreement for Manchester dataset.

**Figure 29:**
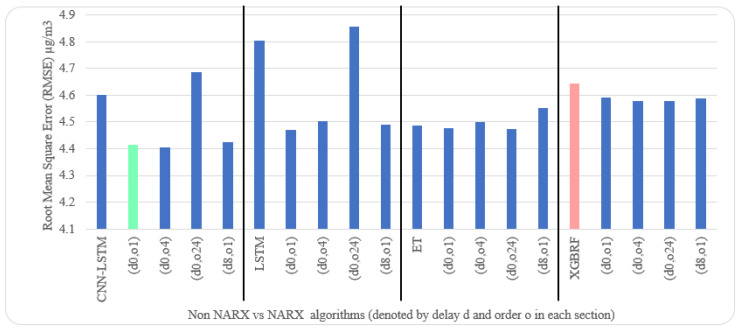
Evaluation results of non-NARX and NARX in terms of root mean square error for the Manchester dataset.

**Figure 30:**
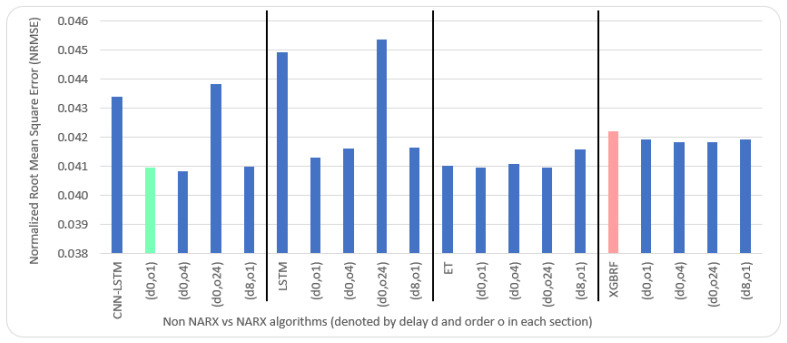
Evaluation results of non-NARX and NARX in terms of normalised root mean square error for the Manchester dataset.

**Figure 31:**
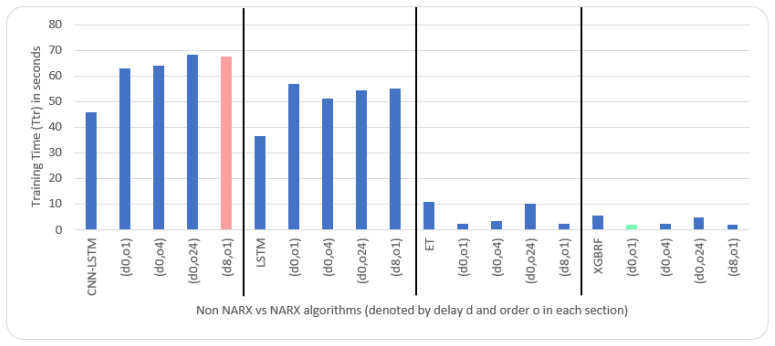
Evaluation results of non-NARX and NARX in terms of offline training time for the Manchester dataset.

**Table 1:** Related work summary.

Reference	Algorithms	Prediction Horizon	Evaluation Metrics	Pros	Cons
[19]	APNet (CNN–LSTM with normalised batching)	Used past 24 h to predict next hour	RMSE, MAE, IA	Viability and usefulness were validated experimentally for predicting PM_2.5_ using their proposal.	Algorithmic forecasts did not precisely follow real trends and were shifted and distorted.
[22]	CNN–LSTM	Used past 24–72 h to predict next 3 h	RMSE, correlation coefficient	Their model is used for processing input from many sites in a city.	They did not verify that their model can be applied to other cities than the one experimented upon.
[23]	CNN–LSTM	Used past 4, 12, and 24 h to predict next hour	MAE, RMSE	They combined data from meteorological and traffic sources and air pollution stations to compare the effectiveness of adding external sources for better air-quality prediction.	They used all the data and features available, which would incur a high computation cost and long execution time.
[24]	Multivariate CNN–LSTM	Used past week to predict next 24 h	MAE, RMSE	CNN obtained air-quality features, decreasing training time; meanwhile, long-term historical input data aided LSTM in the prediction process.	More evaluation metrics could have been applied to verify their models’ performance, stating proximity to actual values such as R2 or IA.
[20]	LSTM	Used past 24 h to predict next hour	RMSE, NRMSE, R^2^, IA	Using NARX minimised data input to a lower limit speeding up the process and improving accuracy in LSTM.	Evaluation using K-Fold is inaccurate.

**Table 2:** Beijing, China dataset statistics.

	PM_2.5_	Cumulated Hours of Rain	Cumulated Wind Speed
**Count**	41,757	43,824	43,824
**Mean**	98.61321	0.194916	23.88914
**Standard Deviation**	92.04928	1.415851	50.01006
**Minimum**	0	0	0.45
**Percentile (25%)**	29	0	1.79
**Percentile (50%)**	72	0	5.37
**Percentile (75%)**	137	0	21.91
**Maximum**	994	36	585.6
**Empty Count**	2067	0	0
**Loss Percentage**	4.95%	0.00%	0.00%
**Coverage Percentage**	95.28%	100.00%	100.00%

**Table 3:** Piccadilly station, Manchester, UK dataset statistics before processing.

	PM_2.5_	M_DIR	M_SPED	M_T	NO	NO_2_	O_3_
**Count**	39,962	42,768	42,768	42,768	42,801	42,710	42,790
**Mean**	10.2795	197.5673	3.3021	9.1598	18.0077	37.2121	28.2244
**Standard Deviation**	10.2253	82.0140	1.8266	5.6743	29.9828	18.2559	19.3880
**Minimum**	−4	0.1	0	−6.9	0	1.5181	0.0998
**Percentile (25%)**	4.3	138.9	1.9	5.2	3.3162	22.9991	11.8744
**Percentile (50%)**	7.6	205.4	2.9	8.9	8.1880	34.7902	26.4430
**Percentile (75%)**	13.1	258.1	4.4	13.1	19.7654	49.0941	41.8099
**Maximum**	404.3	360	13.8	30.6	671.7575	256.1077	138.5515
**Empty Count**	3862	1056	1056	1056	1023	1114	1034
**Loss Percentage**	8.81%	2.41%	2.41%	2.41%	2.33%	2.54%	2.36%
**Coverage Percentage**	91.19%	97.59%	97.59%	97.59%	97.67%	97.46%	97.64%

**Table 4:** Piccadilly station, Manchester, UK dataset statistics after imputation and processing.

	PM_2.5_	M_DIR	M_SPED	M_T	NO	NO_2_	O_3_
**Count**	43,824	43,824	43,824	43,824	43,824	43,824	43,824
**Mean**	10.4240	197.6653	3.3172	9.1687	17.9776	37.3214	28.2042
**Standard Deviation**	9.7384	81.0646	1.8110	5.6170	29.6567	18.1270	19.2519
**Minimum**	0	0.1	0	−6.9	0	1.5181	0.0998
**Percentile (25%)**	4.8	141.6	1.9	5.3	3.4017	23.2407	12.0241
**Percentile (50%)**	7.9	205.6	3	9	8.4868	34.9435	26.4929
**Percentile (75%)**	12.7940	256.6	4.4	13	19.8489	49.2579	41.6438
**Maximum**	404.3	360	13.8	30.6	671.7575	256.1077	138.5515

**Table 5:** Prediction evaluation metrics averaged for Timeseries K-Fold = 10 for the Beijing dataset.

No	Algorithm Name	R^2^ ↑	IA ↑	RMSE (µg/m^3^) ↓	NRMSE ↓	T_tr_ (Seconds) ↓
1	**CNN–LSTM**	0.93151	0.98237	23.22744	0.03776	31.83709
2	(d0, o1)	**0.93498**	0.98304	**22.56670**	**0.03670**	33.48102
3	(d0, o4)	0.93358	0.98264	22.88752	0.03715	31.94185
4	(d0, o24)	0.93136	0.98185	23.23515	0.03780	30.90029
5	(d8, o1)	0.93472	**0.98309**	22.60365	0.03677	*34.92095*
6	**LSTM**	0.93000	0.98157	23.45492	0.03817	23.10278
7	(d0, o1)	0.93372	0.98266	22.81122	0.03709	24.75054
8	(d0, o4)	0.93329	0.98270	22.86120	0.03719	24.73670
9	(d0, o24)	0.92800	0.98108	23.77952	0.03870	23.65251
10	(d8, o1)	0.93119	0.98220	23.30740	0.03764	25.30951
11	**ET**	0.92624	0.98027	24.21871	0.03926	3.86640
12	(d0, o1)	0.92583	0.98018	24.27789	0.03936	1.67357
13	(d0, o4)	0.92609	0.98028	24.21005	0.03927	1.97124
14	(d0, o24)	0.92633	0.98030	24.15589	0.03921	4.09777
15	(d8, o1)	0.92482	0.97992	24.43607	0.03964	1.75481
16	**XGBRF**	*0.92051*	*0.97881*	*25.32772*	*0.04087*	1.39812
17	(d0, o1)	0.92106	0.97893	25.24395	0.04061	**0.90726**
18	(d0, o4)	0.92137	0.97904	25.19564	0.04058	0.98165
19	(d0, o24)	0.92124	0.97901	25.21104	0.04064	1.70556
20	(d8, o1)	0.92116	0.97897	25.22721	0.04060	1.11933
21	APNet [19]	N/A	0.97831	24.22874	N/A	N/A
22	NARX LSTM (d8, o1) [20]	0.9291	0.98150	23.64560	0.03750	15.518

**Table 6:** Prediction evaluation metrics averaged for Timeseries K-Fold = 10 for Manchester, UK dataset.

No	Algorithm Name	R^2^ ↑	IA ↑	RMSE (µg/m^3^) ↓	NRMSE ↓	T_tr_(Seconds) ↓
1	**CNN–LSTM**	0.73343	0.91014	4.60168	0.04338	45.65250
2	(d0, o1)	0.75676	0.92043	4.41522	0.04093	62.93308
3	(d0, o4)	**0.75719**	**0.92129**	**4.40502**	**0.04082**	63.98762
4	(d0, o24)	0.72561	0.90614	4.68568	0.04383	*68.29444*
5	(d8, o1)	0.75587	0.92121	4.42376	0.04098	67.65048
6	**LSTM**	0.71410	0.90178	4.80527	0.04494	36.36427
7	(d0, o1)	0.75132	0.91719	4.46954	0.04131	56.96864
8	(d0, o4)	0.74757	0.91746	4.50223	0.04162	51.03376
9	(d0, o24)	*0.70886*	*0.89991*	*4.85817*	*0.04536*	54.52106
10	(d8, o1)	0.74860	0.91608	4.48958	0.04164	55.04288
11	**ET**	0.75236	0.91677	4.48561	0.04100	10.69692
12	(d0, o1)	0.75413	0.91787	4.47682	0.04096	2.29273
13	(d0, o4)	0.75144	0.91707	4.50112	0.04108	3.35555
14	(d0, o24)	0.75453	0.91775	4.47306	0.04095	10.22563
15	(d8, o1)	0.74594	0.91575	4.55117	0.04158	2.41416
16	**XGBRF**	0.73285	0.91280	4.64339	0.04220	5.45900
17	(d0, o1)	0.74011	0.91516	4.59084	0.04192	1.77346
18	(d0, o4)	0.74169	0.91564	4.57746	0.04181	2.10689
19	(d0, o24)	0.74247	0.91579	4.57905	0.04183	4.59505
20	(d8, o1)	0.74069	0.91578	4.58845	0.04193	**1.71298**

**Table 7:** An excerpt from Beijing dataset matching the sharp transition in results (cv = calm and variable, NW = northwest).

Timestep	Date and Time	PM_2.5_	Cumulated Wind Speed	Combined Wind Direction
30126	9 June 2013 5:00	130	1.78	cv
30127	9 June 2013 6:00	153	2.23	cv
30128	9 June 2013 7:00	110	1.79	NW
30129	9 June 2013 8:00	21	3.58	NW
30130	9 June 2013 9:00	14	9.39	NW
30131	9 June 2013 10:00	13	17.44	NW
30132	9 June 2013 11:00	36	23.25	NW
30133	9 June 2013 12:00	14	29.06	NW

**Table 8:** Output statistics of CNN–LSTM and LSTM along with NARX vs. training and testing output for the seventh iteration for the Beijing dataset.

Testing Count = 3722	Mean	SD	Min	Percentile						Max
				(25%)	(50%)	(75%)	(95%)	(99%)	(99.99%)	
**Training**	101.9	95.1	0	29	75	144	289	434	915.5	994
**Testing**	78	56	4	37	66	107.3	182	257.3	459.2	466
**CNN–LSTM**	77.5	53.8	4	36	66	107	179	248.6	379.7	382
(d0, o1)	**78.4 **	**54.9**	**4**	**37**	**67**	**108**	**182**	**255.3**	**396.7**	**399**
(d0, o4)	78.2	53.5	5	38	67	107	178	248	383.6	387
(d0, o24)	77.1	52.7	**−3.0**	37	66	106	176.5	247.9	363.5	365
(d8, o1)	78.8	55.1	**−15.0**	38	67	108	182	254.6	400.4	403
**LSTM**	78.2	53.8	**−7.0**	36	66	107	181.5	255.6	359.2	360
(d0, o1)	77.8	52.8	**−7.0**	38	67	107	177	246.3	368.1	370
(d0, o4)	77.1	53.1	**−11.0**	36	66	106	177	248	361.7	364
(d0, o24)	77	51.8	**−12.0**	36	67	106	174	247.3	357.1	359
(d8, o1)	77.8	52.8	8	38	67	107	177	244.3	368	371

**Table 9:** Output statistics of Extra Trees and XGBRF along with NARX vs. the training and testing output for the seventh iteration for the Beijing dataset.

Testing Count = 3722	Mean	SD	Min	Percentile						Max
				(25%)	(50%)	(75%)	(95%)	(99%)	(99.99%)	
**Training**	101.9	95.1	0	29	75	144	289	434	915.5	994
**Testing**	78	56	4	37	66	107.3	182	257.3	459.2	466
**ET**	79.5	54.2	**5**	39	69	108	179.5	252	418.2	**422**
(d0, o1)	79.6	54.7	**6**	39	69	108	179.5	254.2	451.5	** 473 **
(d0, o4)	79.6	54.7	**5**	39	69	107	179.5	253.3	439.7	**442**
(d0, o24)	79.6	54.4	**5**	39	69	109	180	253	424.2	**425**
(d8, o1)	79.6	55	**5**	39	69	108	181	254.3	447.9	**449**
**XGBRF**	79.4	54.5	**10**	39	71	105	178	252.3	442.3	**448**
(d0, o1)	79.4	54.7	**10**	39	70	105	178	251.3	449	**455**
(d0, o4)	79.4	54.6	**9**	39	70	105	178	251.3	449.3	**455**
(d0, o24)	79.4	54.6	**10**	39	70	105	178	252.3	446.7	**452**
(d8, o1)	79.4	54.7	**10**	39	70.5	105	178.5	252	449	**455**

**Table 10:** Output statistics of CNN–LSTM and LSTM along with NARX vs. training and testing output for the seventh iteration for the Manchester dataset.

Testing Count = 3960	Mean	SD	Min	Percentile						Max
				(25%)	(50%)	(75%)	(95%)	(99%)	(99.99%)	
**Training**	10	9.7	0	4.5	7.5	12.3	27.8	45.2	253.9	404.3
**Testing**	11.8	10.1	0	6	9	15	28	48.4	131	135
**CNN–LSTM**	11.3	7.9	**−1.0**	6	9	15	26	40	65.4	67
(d0, o1)	11.1	7.7	**−3.0**	6	9	14	25	40	66	66
(d0, o4)	**11.4**	**7.9**	**−1.0**	**6**	**9**	**15**	**26**	**40**	**65**	**65**
(d0, o24)	11.1	7.4	**−2.0**	6	9	14	25	38	59.6	60
(d8, o1)	11.3	7.6	**−1.0**	6	9	14	26	39.4	66	66
**LSTM**	11.4	7.6	**−2.0**	6	9	14	26	41	61.4	63
(d0, o1)	11.6	8.1	**−3.0**	6	9	14	26	42	78.4	80
(d0, o4)	11.2	8	**−1.0**	6	9	14	26	41	71.4	73
(d0, o24)	11.5	7.6	**−3.0**	6	9	15	26	40	66.6	67
(d8, o1)	11.3	8.4	**−4.0**	6	9	14	27	43	73.8	75

**Table 11:** Output statistics of Extra Trees and XGBRF along with NARX vs. the training and testing output for the seventh iteration for the Manchester dataset.

Testing Count = 3960	Mean	SD	Min	Percentile						Max
				(25%)	(50%)	(75%)	(95%)	(99%)	(99.99%)	
**Training**	10	9.7	0	4.5	7.5	12.3	27.8	45.2	253.9	404.3
**Testing**	11.8	10.1	0	6	9	15	28	48.4	131	135
**ET**	11.4	8.5	**2**	6	9	14	26	43	113.7	**120**
(d0, o1)	11.4	8.5	**2**	6	9	15	26	40	110.8	**112**
(d0, o4)	11.5	8.7	**2**	6	9	15	26	42	140	** 142 **
(d0, o24)	11.4	8.5	**2**	6	9	15	26	40	122.9	**130**
(d8, o1)	11.6	8.8	**2**	6	9	15	27	43	130.1	** 138 **
**XGBRF**	11.6	9.8	**3**	6	9	14	27	46.4	189.2	** 190 **
(d0, o1)	11.6	9.4	**3**	6	9	14	27	46	157.2	** 158 **
(d0, o4)	11.5	9.3	**3**	6	9	14	27	45.4	147.2	** 148 **
(d0, o24)	11.5	9.2	**3**	6	9	14	27	46	139.6	** 140 **
(d8, o1)	11.6	9.4	**3**	6	9	14	27	46	157.2	** 158 **

## Data Availability

Publicly available datasets were analysed in this study. Beijing dataset was obtained from https://archive.ics.uci.edu/ml/datasets/Beijing+PM2.5+Data accessed on 2 March 2022 and Manchester dataset was obtained from https://uk-air.defra.gov.uk/data/data_selector_service accessed on 2 March 2022.
